# Supplementary material for: Investigation of Imidazolinone Herbicide Resistance Gene with KASP Markers for Japonica/Geng Rice Varieties in the Huanghuaihai Region of China
Source: Plants (Basel). 2024 Apr 14;13(8):1097. doi: 10.3390/plants13081097 (PMC11053791; doi:10.3390/plants13081097)
Supplement: Supplementary file 1 [file Figure S1.pdf › Figure S1.pdf]

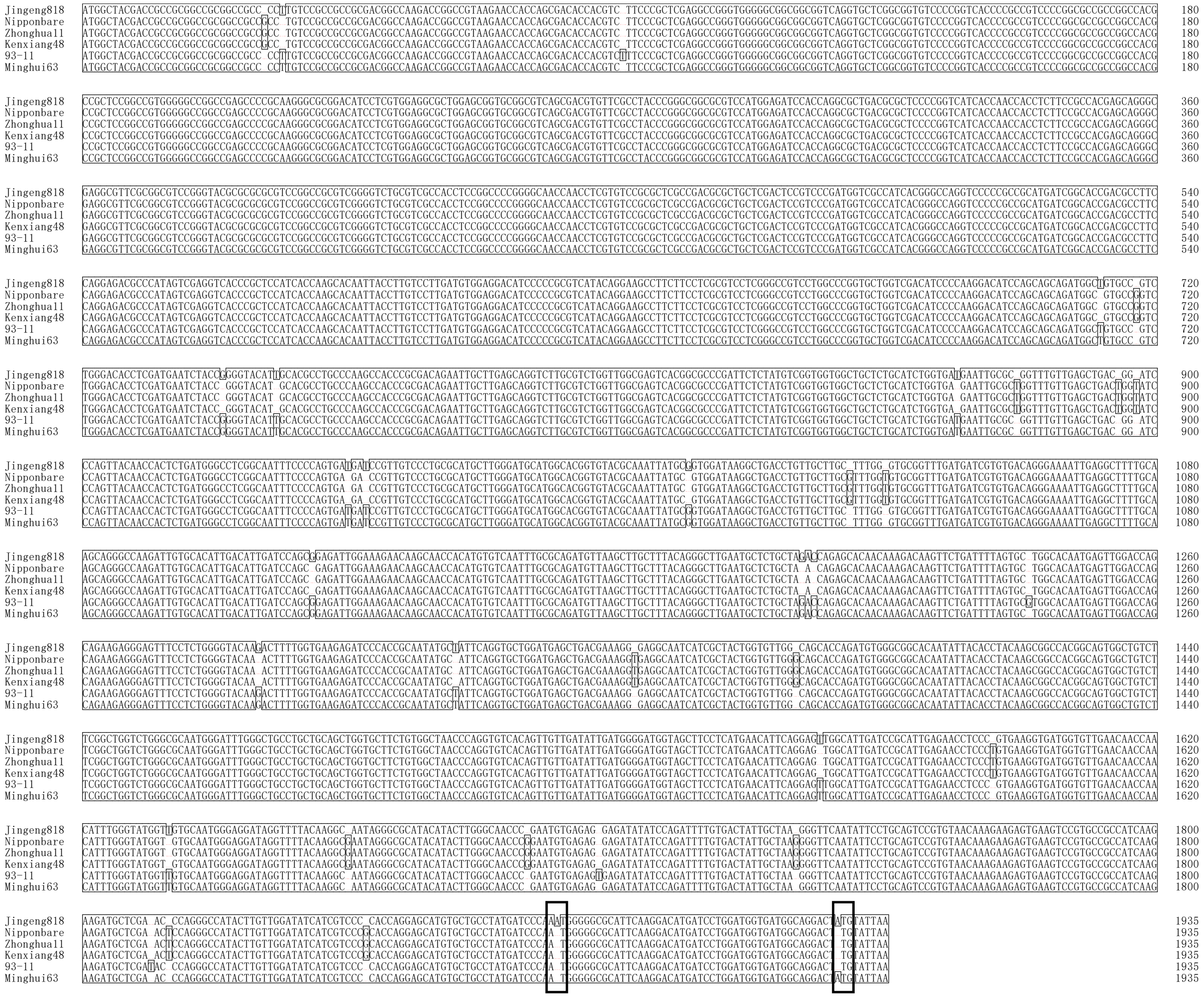

**Figure S1.** Sequence alignment of *OsALS1* from Jingeng 818, Nipponbare, Zhonghua11, Kenxiang 48, 93-11 and Minghui 63.
